# Inequalities and identity processes in crises: Recommendations for facilitating safe response to the COVID‐19 pandemic

**DOI:** 10.1111/bjso.12400

**Published:** 2020-06-25

**Authors:** Anne Templeton, Selin Tekin Guven, Carina Hoerst, Sara Vestergren, Louise Davidson, Susie Ballentyne, Hannah Madsen, Sanjeedah Choudhury

**Affiliations:** ^1^ University of Edinburgh UK; ^2^ University of Sussex UK; ^3^ University of Salford UK

**Keywords:** Identity processes, behaviour change, social norms, social identities, inequality, societal inequality, leadership, collective actions, coronavirus, COVID‐19

## Abstract

Structural inequalities and identity processes are pivotal to understanding public response to COVID‐19. We discuss how identity processes can be used to promote community‐level support, safe normative behaviour, and increase compliance with guidance. However, we caution how government failure to account for structural inequalities can alienate vulnerable groups, inhibit groups from being able to follow guidance, and lead to the creation of new groups in response to illegitimate treatment. Moreover, we look ahead to the longitudinal impacts of inequalities during pandemics and advise government bodies should address identity‐based inequalities to mitigate negative relations with the public and subsequent collective protest.

## Background

Identity processes related to structural inequalities are fundamental to understanding how and why disasters should be managed. During public health emergencies, members of racial and ethnic minority groups have higher rates of both illness and death (Dash, 2013). In the COVID‐19 pandemic, BAME (Black, Asian, and minority ethnic) people are more likely to die from COVID‐19 than White populations in both the United States (Garg *et al*., 2020) and United Kingdom (ONS, 2020). Following guidance such as physical distancing and protecting elderly populations could be more difficult for BAME groups as they are more likely to live in densely populated areas (Bravo *et al*., 2018; Jackson *et al*., 2000) and in multi‐generational households (Loftquist, 2012), thus limiting the possibility to protect older generations. While facing these institutional barriers, BAME people are also 54% more likely to be fined under COVID‐19 rules for not following guidance (Gidda, 2020). Despite high willingness to follow guidance to self‐isolate, people from the low‐socioeconomic status (SES) households are six times less likely to be able to work from home and three times less likely to be able to self‐isolate (Atchison *et al*., 2020). These inequalities have led to calls for BAME and low‐SES populations to receive additional financial aid (e.g., Ubele, 2020), protective measures, and mental health support (e.g., NHS, 2020) to mitigate the disproportionate effect of COVID‐19 on the communities.

Research on collective behaviour demonstrates how new identities can act as a protective buffer for well‐being during crises (Alfadhli *et al*., 2019), how identity‐relevant social norms regarding safety can facilitate behaviour change and social support at a collective level (Drury & Reicher, 2009), and how leaders can act as effective emblems of desired behaviour (Platow *et al*, 2015). However, research also demonstrates how social norms can encourage unsafe behaviours (e.g., Hopkins & Reicher, 2016; Stott *et al*., 2001), how leaders can alienate groups by treating them illegitimately (Haslam *et al*., 2010) and how identity‐based structural inequalities decrease community cohesion, compliance with guidance, and perceived legitimacy of governments (Mazepus & van Leeuwen, 2019). To manage disasters effectively, governments and other state actors must seek to understand and address the effects that identity‐based structural inequalities have on community cohesion and support, ability to follow government guidance, perception of leaders, and community activism post‐pandemic. We explore these research avenues in turn and focus on providing guidance to improve community well‐being, encourage social support, facilitate safe behaviour, provide legitimate leadership, maintain community cohesion post‐pandemic, and mitigate resentment towards governments post‐disaster. A summary of key recommendations is provided in Table 1.

**Table 1:** Summary of recommendations for governments, state actors, and other stakeholders to effectively handle pandemics

Overarching theme	Recommendations	Actions
Facilitate emergent groups and mobilize social identities	Encourage group‐based support	Mobilize neighbourhood identities, prime the importance of looking after neighbours for the good of the group.
	Mitigate division by addressing societal inequalities	Coordinate collective support networks that address additional support needed by BAME and low‐SES communities.
Make safe behaviour normative and inclusive	Create new norms within existing groups	Demonstrate how desired behaviour aligns with the group definition and group interest.
	Address societal inequalities	Create norms that the public can follow in order to avoid outgroup exclusion.
		Provide resources to facilitate following guidance, for example, increase resources and work with housing organizations and community clinics.
		Work with local community leaders and groups.
Provide legitimate leadership	Provide clear information	Provide guidance that: respects the needs of communities; explains why behaviour is necessary; informs how to follow guidelines; provides regular updates about actions being taken by authorities.
	Provide identity‐based leadership	Act in the group interest and follow the guidance given to the public.
Facilitate maintenance of new communities	Maintain community support	Encourage events and activities that enable people to maintain their social connections.
	Address inequalities now	Identify affected groups within communities to ensure they receive sufficient long‐term support (e.g., financial aid).
New avenues for social identities and collective behaviour research	Social norm processes	Research how organizations can change pre‐existing norms to encourage new safe normative behaviours.
	Learn from other disciplines	Incorporate research on inequalities in mass disasters, e.g., how different groups are affected by disasters, prejudice groups face when they are unable to comply with guidance, and how illegitimate treatment can lead to long‐term campaigns.

## Facilitate inclusive emergent support groups

People have multiple social identities (Tajfel & Turner, 1979), for example as a member of their family or nation. Previous research on disasters suggests that new social groups can form when people become united under a shared social identity through a common fate (Drury *et al*., 2019; Drury *et al*., 2009). In line with self‐categorization theory (Turner *et al*., 1987), people may shift from their personal identities to a shared identity as a member of the particular group facing the disaster. This is important for understanding social support since people are more likely to provide help to ingroup, rather than outgroup, members in emergencies (e.g., Levine *et al*., 2005), and the belief that ingroup members will provide help can lead to increased engagement in supportive actions for the community (e.g., Drury *et al*., 2016). As such, these shared social identities can be harnessed to mobilize collective support within communities (e.g., see Vestergren *et al*., 2018).

However, previous research on emergencies has mostly focussed on situations where survivors shared an equal threat. When people believe they are disproportionately affected based on an existing social identity (e.g., being a traveller, see Ntontis *et al*., 2020), it can make that identity salient and lead to construing the situation in terms of intergroup relations and pre‐existing discrimination, thus hindering identification with the new disaster group. Moreover, new social identities can emerge based on shared reactions to a situation (Thomas & McGarty, 2009; Thomas *et al*., 2009, 2012) such as that BAME and low‐SES populations are disproportionately affected by the pandemic. When people define themselves as groups based around shared opinions, it can be a strong predictor of commitment to social actions regarding that identity (Bliuc *et al*., 2007). Thus, governments that coordinate public response based on assumptions of equal threat risk creating further divisions. Instead, governments should mitigate divisions by coordinating collective support networks that address additional support needed by BAME and low‐SES communities.

## Create inclusive norms for safe behaviour

To facilitate safe public response in the COVID‐19 pandemic, governments and state actors should look to how group definitions and social norms can be harnessed to promote particular behaviour. Group members have shared understandings of their group definitions and what behaviours are considered normative, beneficial, or disadvantageous for the group (McGarty, 1999; Tajfel & Turner, 1979). This can both promote *and* reduce safe behaviours during the COVID‐19 pandemic. For example, predefined notions around masculinity can deter men from taking safety precautions such as wearing masks due to believing their group to be too strong to become ill (Capraro & Barcelo, 2020; Levita, 2020). Creating new norms (e.g., physical distancing) within existing social identities and limiting previous norms that are now dangerous (e.g., handshaking) also poses a unique challenge post‐COVID‐19 as people return to familiar environments that hold expected behaviours (e.g., physical closeness). To make safe behaviour normative, safe behaviours should be encouraged and unsafe behaviours discouraged in the group interest, and collective safety made central to group definition. For example, the Prime Minister of New Zealand, Jacinda Ardern, addressed the nation emphasizing a group definition that New Zealanders are community‐minded and look after one another. In the same address, Ardern emphasized the common threat of the pandemic to New Zealand and said that actions from friends, family, and neighbours were needed to support vulnerable populations (RNZ, 2020). Importantly, the address was accompanied by clear government guidance about how to interact with community members safely (New Zealand Government, 2020).

When group members know which behaviours are normative (safe) and non‐normative (unsafe), they can collectively self‐regulate unsafe behaviours by encouraging ingroup members to follow established norms (e.g., Drury *et al*., 2015; Stott *et al*., 2001). However, following social norms depends on the perception that the action is within the shared goals of the group (Ntontis *et al*., 2020). Social norms must be actively established to encourage behavioural transference during the COVID‐19 pandemic. For example, maintaining physical distance from our ingroup provides a unique challenge as we tend to seek greater physical proximity to ingroup, rather than outgroup, members, and feel safe and take joy from it (Novelli, 2010; Novelli *et al*., 2013). Making distancing normative can be achieved through demonstrating that it is within the shared goals of the group, e.g., collectively mitigating virus spread. Thus, to facilitate community support and adherence to guidelines, governments should promote a shared social identity which defines safe health behaviours as normative, in the groups’ interest, and under shared goals.

A caveat when encouraging collective safe behaviour is that compliance with guidance can be challenging when social identities are incongruent with the ability to adhere. Citizens may want to follow official guidance but be unable to adhere due to group‐based inequalities. For example, physical distancing is difficult for BAME groups who live in densely populated areas (Jackson *et al*., 2000) and are highly represented in roles that require being close to others and are associated with increased mortality rates during the COVID‐19 pandemic (Public Health England, 2020) such as being key workers (The Health Foundation, 2020) and transport drivers (ONS, 2020). Previous research also highlights that BAME and low‐socio‐economic groups are vulnerable to unstable working conditions in disasters (Wang *et al*., 2017) which can make it difficult to follow guidance such as staying home. In the United Kingdom, BAME groups are more likely to be unemployed than the White population (Gov.uk, 2019), and low‐SES groups are most likely to experience disruption to essential services like food banks (Bulman, 2020). As a result, people from low‐SES backgrounds may be forced to leave their home for financial reasons (Atchison *et al*., 2020; Barnard, 2020), yet are often the most overlooked in emergency planning (Blake *et al*, 2017; Pelling & Dill, 2006; Smith, 2006).

Creating societal norms and guidance that ignore structural societal inequality by including infeasible practices (e.g., isolating) risks exclusion of vulnerable groups. These seemingly non‐normative behaviours (e.g., leaving home) can lead to punishment such as BAME populations being disproportionately fined for not following guidance (Gidda, 2020). It can also lead to exclusion from other groups due to being blamed for the disease (e.g., blaming Muslims in India for COVID‐19 spread, see Dovidio *et al*., 2020). Consequently, this can increase the risk of being perceived as an outgroup compared to those united against the virus. In turn, being rendered outgroup can increase the animosity, prejudice, and hostility already faced (Jackson *et al*., 2019), and decrease protective efforts through reduced empathy towards those groups (Cikara *et al*., 2011).

To increase inclusivity and promote safe behaviour in vulnerable communities during disasters, governments can increase resources for community‐based organizations such as housing organizations supporting at‐risk tenants experiencing financial difficulties (Shelter, 2020). These community‐based organizations can effectively support vulnerable people within communities (e.g., Radical Housing Network *et al*., 2019) and are likely to be more trusted since they are within the community’s shared social identity. These organizations can support vulnerable communities to adhere to guidelines, which in turn can decrease the risk of antagonist and hostile intergroup relations.

Inequalities in disasters have been studied by various disciplines such as geography, history, anthropology, and sociology (e.g., Oliver‐Smith, 1991; Pitarka *et al*., 1998; Tierney *et al*., 2006). However, the implications of social inequality in mass disasters have received little attention within the social identity literature (for exceptions, see Drury & Tekin Guven, 2020; Muldoon *et al*., 2017). Social identity research can learn from other disciplines regarding the role of structural inequalities in responses to mass disasters. Specifically, the extent to which people can follow guidance given by more privileged groups and how disasters disproportionately affect people from different social backgrounds can develop our understanding of intergroup and intragroup relations in crises.

## Promote behaviour change through legitimate leadership

The public are more likely to adhere to government behavioural recommendations when they trust the government can control the virus spread (Rubin *et al*., 2009; Tang & Wong, 2003). Successful leadership (i.e., promoting legitimacy and public adherence to recommendations) hinges on public perception that leaders are part of the group and working in the group interest (Platow *et al*., 2015; Reicher, 1984; Renwick, 2019). Actions of leaders during the pandemic have showcased how they can successfully facilitate behaviour change, but conversely how leaders can create divisions by acting differently to those they guide and alienate group members through their actions.

To lead effectively, leaders must be seen as representatives of their group in order to define shared norms, which entails being seen as a prototypical member and acting in the group’s interest (Haslam *et al*., 2010; Steffens *et al*., 2013). The way that Jacinda Arden positions herself as a member of the national group and advocates that new behaviours are in the national interest can serve as a successful example of leaders using identity principles to facilitate behaviour change (see Wilson, 2020). However, some actions by leaders demonstrate how divisions can be created by disobeying guidance the public are required to follow, such as Donald Trump refusing to wear a facemask despite his administration’s advice to the public (Smith, 2020) or Dominic Cummings travelling 250 miles (Reicher, 2020). Actions seen to undermine government guidance risks reducing public trust and legitimacy of government leadership by creating different systems of behaviour for the government and the public, thus eroding the effectiveness of future leadership guidance (Pucic, 2014).

Similarly, governments can alienate groups by failing to communicate appropriately or provide adequate resources. Governments can increase their perceived legitimacy by respecting the needs of communities, providing health‐focused information about why procedures are necessary, providing regular updates about government actions, and giving sufficient practical information about how to respond (Bonell *et al*., 2020; Carter *et al*., 2016). Crucially, governments should follow fair procedures and distribute information and resources appropriately (Mazepus & van Leeuwen, 2019), since social disparity in who receives help from state actors can exacerbate societal divisions when those most disadvantaged are neglected (Gratz, 2015; Hartman & Squires, 2006). To avoid these failures, government bodies should create plans for communicating with local groups and provide them with necessary resources so that structural inequalities are prioritized and suitably managed. This would ensure that the most vulnerable populations receive sufficient help to counteract their disadvantaged positions and make it possible to adhere to guidance.

## Maintain societal cohesion post‐disaster by addressing inequalities

Post‐pandemic, communities can provide vital support systems that improve well‐being. However, the breakdown of support systems can leave vulnerable groups stranded, and common experiences of illegitimate treatment during the pandemic can be mobilized into collective actions against authorities.

Maintaining supportive communities will be critical to improving well‐being post‐pandemic. Social identification with groups can improve well‐being during stressful periods when support is perceived (Haslam *et al*., 2010; Jetten *et al*., 2012), and perceived collective efficacy can increase positive psychological outcomes such as post‐traumatic growth (Muldoon *et al*., 2017). Community responses to flooding (Ntontis *et al*., 2020) and environmental campaigns (Vestergren *et al*., 2018, 2019) demonstrate how emergent groups can be sustained or diminished through mutual recognition of disaster experiences, communication, and participation in collective events related to the disaster. To maintain supportive communities after COVID‐19, governments can encourage collective events based around the new supportive communities to maintain the new identities and social connections, such as continuing resource sharing and providing support within communities.

In contrast, social identities can break down and lead to individualist behaviours if there is no longer a perceived common fate (Ntontis *et al*., 2020) or no possibilities for intragroup relations to endure (Vestergren *et al*., 2018). This is particularly important for BAME and low‐SES populations as they are more likely to suffer mental health and financial difficulties in the aftermath of disasters (de Silva & Kawasaki, 2018; Jones *et al*., 2011), yet risk losing support from people who no longer perceive COVID‐19 as a collective issue. One way to mitigate this is for governments to provide additional financial and mental health support to mitigate the disproportionate effect of COVID‐19 on the communities. However, different communities may have specific needs, and mistrust between disaster‐affected populations and government representatives can lead to the community needs not being met (e.g., Legerski *et al*., 2012). As such, government bodies should seek to work with community members to identify needs and provide resources. This can be done by working with trusted community leaders and providing resources to already established community initiatives (e.g., community clinics) who are trusted and whom people turn to for support (for successful examples, see Charles, 2019).

Trust in authorities is also integral to understanding collective reactions in the aftermath of disasters. The perception that responding agencies have acted unfairly can create enduring animosity and render them as outgroups (Vestergren *et al*., 2018). This can make it more difficult for governments to facilitate public adherence to guidelines and the support needed in the aftermath of the pandemic. In certain circumstances, those affected by disasters develop collective campaigns to hold authorities accountable for issues such as inadequate resources (Mazepus & van Leeuwen, 2019) and services (Aldrich, 2013; Lewis, 2005). For example, campaigns such as protests against resource inequality and lack of government communication in the Ebola outbreak in Liberia, Guinea, and Sierra Leone, resulted in violent clashes between groups of the public and government forces (Cohn & Kutalek, 2016) and serve as a warning to where disaster mismanagement can lead.

To avoid long‐term public opposition and collective campaigns, governments and state actors should empower groups by recognizing inequalities and disadvantages, distributing resources fairly, and providing adequate economic aid and government support. Rather than imposing measures with which people cannot comply (e.g., staying home when one cannot afford to) and imposing inadequate top‐down support, governments should focus on cooperating with groups and listening to their needs then provide them accordingly.

## Conclusions

Government handling of the COVID‐19 pandemic has shone a light on social identity research and exposed avenues for future work. Research on social identities is crucial to understanding community resilience, facilitating safe social norms, and providing effective leadership in pandemics. Importantly, the research indicates how identity definitions and norms can be counterproductive to safety, as well as how poor leadership can exasperate inequalities and lead to long‐term challenges to governance.

We argue that future research should focus on the importance of inequalities in understanding response to mass disasters. To manage disasters safely, it is imperative that governments provide fair and legitimate support to mitigate stigmatization of marginalized groups. Addressing systematic inequalities should be fundamental to disaster planning to improve access to resources and opportunities for compliance, and avoid animosity during and post‐pandemic. Governments and state actors should see communities as an additional resource for collective social support, but they must empower groups through giving them sufficient resources to help overcome existing inequalities. The importance of these areas in mass disasters is often overlooked in the social identity literature. Thus, we recommend that social identity theorists should look to research from other disciplines in mass disasters to enhance understanding of intergroup relations and actions against inequalities.

## Author contribution

Anne Templeton (Conceptualization; Writing – original draft; Writing – review & editing) Selin Tekin Guven (Conceptualization; Writing – review & editing) Carina Hoerst (Conceptualization; Writing – review & editing) Sara Vestergren (Conceptualization; Writing – review & editing) Louise Davidson (Conceptualization; Writing – review & editing) Susie Ballentyne (Conceptualization; Writing – review & editing) Hannah Madsen (Conceptualization; Writing – review & editing) Sanjeedah Choudhury (Conceptualization; Writing – review & editing)

## Conflict of interest

All authors declare no conflict of interest.

## Data Availability

Data sharing is not applicable to this article as no new data were created or analysed in this study.
